# Supplementary material for: Supervised learning is an accurate method for network-based gene classification
Source: Bioinformatics. 2020 Apr 14;36(11):3457–65. doi: 10.1093/bioinformatics/btaa150 (PMC7267831; doi:10.1093/bioinformatics/btaa150)
Supplement: btaa150_Supplementary_Data [file btaa150_supplementary_data.pdf]

Supervised learning is an accurate method for network-based gene classification

Supplemental Material

Section 1: Methods and Data

Section 1.1: Networks

The networks used in this study are BioGRID, STRING-EXP, InBioMap, GIANT-TN, and STRING. Detailed information about the network properties and sources can be seen in Table S1, with the network construction method and interaction type information coming from (Huang *et al.*, 2018). BioGRID (version 3.4.136) is a low-throughput network that includes both genetic interactions, as well as physical protein-protein interactions (Stark *et al.*, 2006). InBioMap (version 2016_09_12) is a high-throughput, scored network that contains physical protein-protein interactions as well as pathway database annotations incorporated as edges (Li *et al.*, 2017). We used the "final-scores" as the edge weights. STRING (version 10.0) is a high-throughput, scored network that aggregates information from many data sources (Szklarczyk *et al.*, 2015). We used two different STRING networks. First, we used the "combined" network that directly includes database annotations, text-mining, ortholog information, co-expression, and physical protein interactions (referred to as "STRING" in this study). We also used a subset of edges in STRING that had just the "experiments" data, thus restricting the network to one constructed just from physical protein interactions in humans (referred to as "STRING-EXP" in this study). For both networks, we used the corresponding relationship scores as edge weights, after normalizing them to lie between 0 and 1. The GIANT-TN (version 1.0) network is the tissue-naïve network from GIANT (Greene *et al.*, 2015), referred to as the "Global" network on the website, and is constructed from both low- and high-throughput data, and includes information from co-expression, non-protein sources, regulatory data, and physical protein-protein interactions. The GIANT-TN network is a fully connected, scored network. To add sparsity to the GIANT-TN network, we removed all edges with scores below 0.01 (equal to the prior the Bayesian model used to construct the network). It is worth noting here that the purpose of this study is not to compare networks against each other, but rather to determine the performance of SL methods vs LP methods on various types of networks.

Table S1. Information on the molecular networks. LT : low-throughput, HT : high-throughput, G : genetic, P : physical, DA : database annotations, CE : co-expression, NP : non-protein, R : regulation, CC : co-citation, O : orthologous.

Network	Number of Genes	Number of Edges	Edge Density	Network Construction Method	Weighted	Interaction Type
BioGRID	20,558	238,474	1.13e-3	LT	No	G, P
STRING-EXP	14,089	141,629	7.08e-4	HT	Yes	P
InBioMap	17,399	644,862	1.58e-3	HT	Yes	P, DA
GIANT-TN	25,689	38,904,929	1.92e-3	LT, HT	Yes	CE, NP, P, R
STRING	17,352	3,640,737	7.20e-3	HT	Yes	CC, CE, O, DA, P

Section 1.2: Model Selection and Hyperparameter Tuning

The restart hyperparameter α used in generating an influence matrix was determined by doing a grid search over values between 0.55 and 0.95 in 0.1 steps for all networks and geneset collections, optimizing for auPRC using label propagation (Fig. S1). In general, there was not a strong dependence on α. It can be seen in Figure S1 that a higher restart probability resulted in marginally better performance for the larger networks (STRING and GLOBAL), whereas as a smaller restart probability led to nominally better performance for the smaller networks such as BioGRID. In this study, we used $\alpha = 0.85$ for every geneset-collection–network combination, as $\alpha = 0.85$ offered good performance and had low variance. This α was used for both LP-I and SL-I. We stress that the tuning of α was never done for SL-I, and thus, our finding that SL methods generally outperform LP methods is not biased by this parameter tuning.

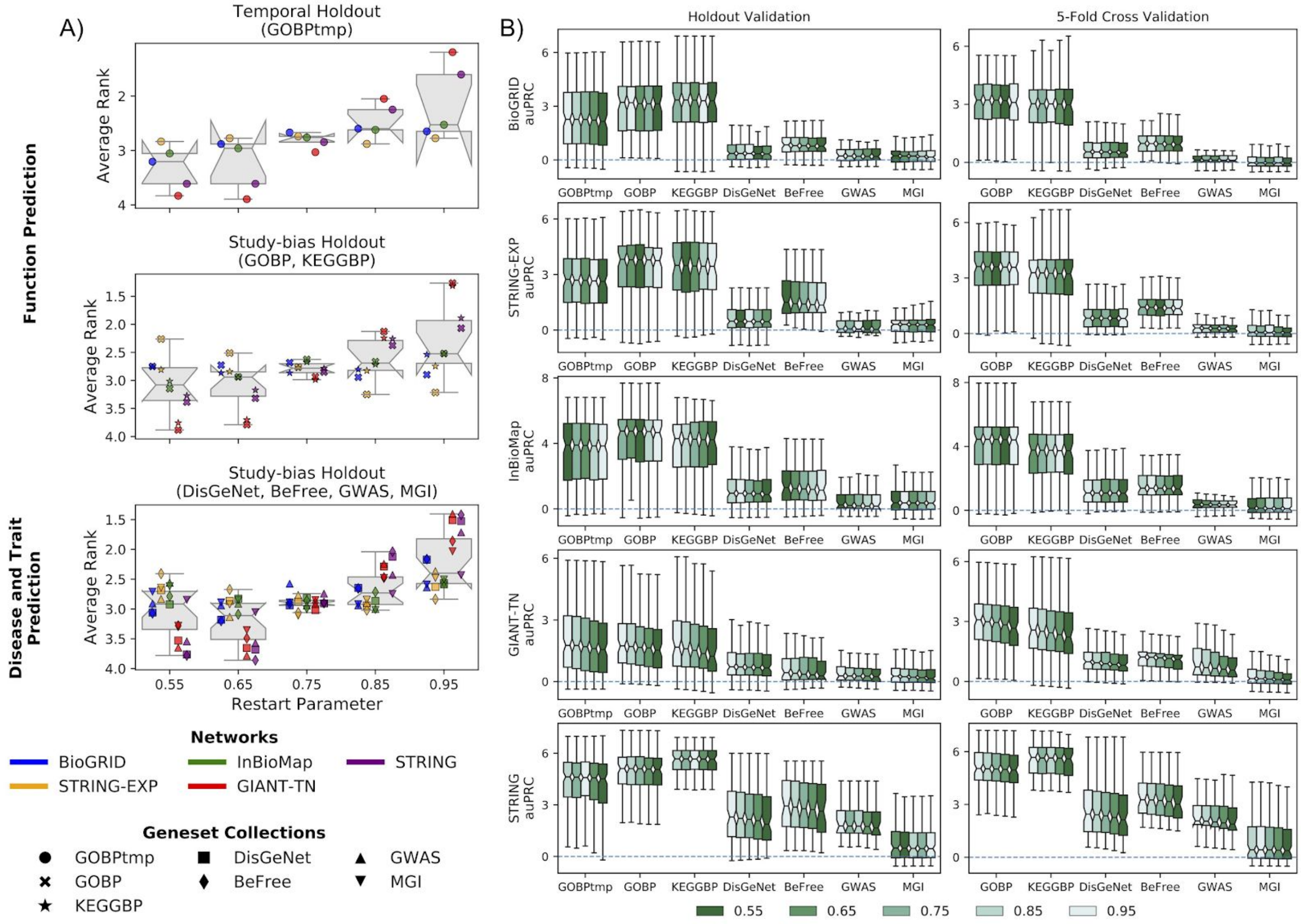

Fig. S1. Tuning the restart probability hyperparameter for label propagation. A) Each point in each boxplot represents the average rank for a geneset-collection–network combination, where the five restart probabilities that were tried were ranked in terms of performance (auPRC) for each geneset in a geneset-collection using the standard competition ranking. A restart probability of 0.85 was chosen for this study as it resulted in good overall performance as well as low variance in performance for the different genset-collection–network combinations. B) The performance for each individual geneset-collection–network combination is compared across the five restart probabilities: 0.55, 0.65, 0.75, 0.85 and 0.95. The methods are ranked by median value of auRPC with the highest scoring method on the left. There is no strong dependence of auRPC on the restart probability.

Model selection of the supervised learning classifier was done by comparing six popular classifiers that are implemented in Python package *Scikit Learn* (Pedregosa *et al.*, 2011). To determine the best supervised learning classifier, we compared their performance over every geneset-collection–network combination using their default hyperparameters in version 0.19 of Scikit Learn (Fig. S2). Logistic regression with L2 regularization is marginally better than linear support vector machines and both these classifiers outperform random forest, logistic regression with L1 regularization, and support vector machines using the radial basis kernel and the 2nd order polynomial kernel. We note that the non-linear SVMs (radial basis and polynomial kernels) took over two orders-of-magnitude longer to train, and thus, those models are not included in the five-fold cross validation results.

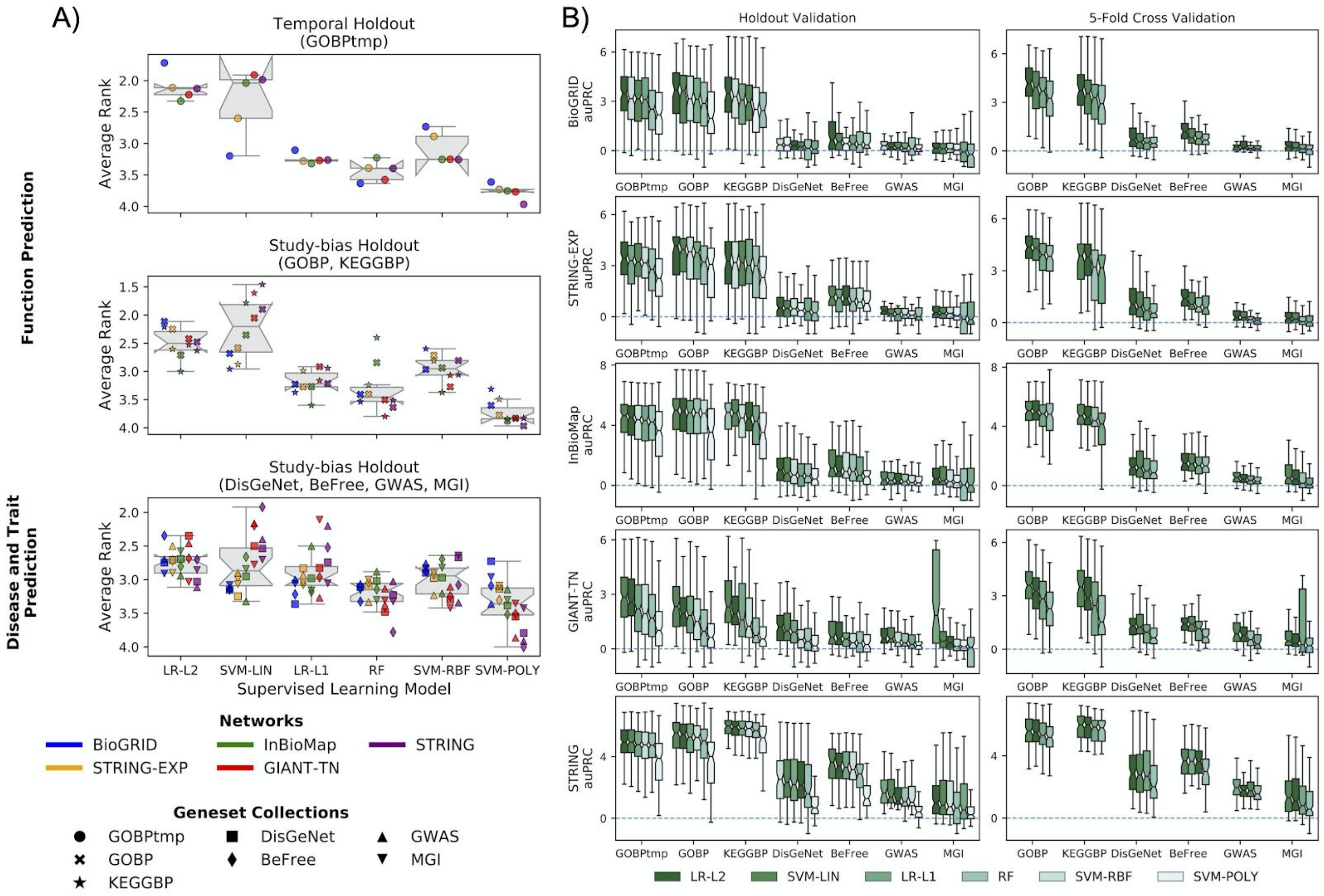

Fig. S2. Comparison of classifiers for supervised learning. A) Each point in each boxplot represents the average rank for a geneset-collection–network combination, where the four classifiers were ranked by the auPRC for each geneset in a geneset-collection using the standard competition ranking. Logistic regression with L2 regularization (LR-L2) was chosen as the classifier for supervised learning as it had slightly better overall performance than a linear support vector machine (SVM). B) The auRPC for each individual geneset-collection–network combination is compared across six supervised learning classifiers: logistic-regression with L1 regularization (LR-L1), LR-L2, SVM models with three kernels (linear; SVM-LIN, radial basis function; SVM-RBF, 2nd order polynomial; SVM-POLY) and a random forest (RF). The classifiers are ranked by median value with the best performing one on the left.

For the model selection of the embedding technique, we chose *node2vec* (Grover and Leskovec, 2016) because its competitive performance and ease of use (Goyal and Ferrara, 2018). The following hyperparameters were tuned based on the aggregated performance across all geneset-collections–network combinations: *p* - the breadth first search parameter, *q* - the depth first search parameter, *d* - embedding size, *l*

- walk length, and *k* - context window size. We left *r* (number of walks per node) at it's default value. Since *p* and *q* are coupled, we performed a grid search for these two parameters leaving all others constant. Each of the other hyperparameters was tuned by leaving the rest at their default values as described in the original *node2vec* publication. The values for the hyperparameters were tuned over are; *p, q* - [0.1. 0.5, 1, 5, 10], *d* - [64, 128, 256, 512, 1024, 2048], *l* - [20, 40, 60, 80, 100, 120, 140, 160, 180, 200], and *k* - [2, 4, 8, 16, 32, 64]. We found that in general there was a large range of values for each parameter where the results were near optimal, and we chose – $p = 0.1$, $q = 0.1$, $d = 512$, $k = 8$, $l = 120$, and $r = 10$ – for every geneset-collection–network combination.

Section 1.3: Geneset-collections

The geneset-collections used in this work are from the Gene Ontology (from version 2 of MyGene.info API with data retrieved on 2018-05-18, GOBPtmp, GOBP) (The Gene Ontology Consortium, 2019; Ashburner *et al.*, 2000; Wu *et al.*, 2013; Xin *et al.*, 2016), Kyoto Encyclopedia of Genes and Genomes (from version 6.1 of MSigDB, KEGGBP) (Kanehisa and Goto, 2000; Kanehisa *et al.*, 2017, 2019), DisGeNet (version 5.0, DisGeNet, BeFree) (Piñero *et al.*, 2017, 2015), GWAS from a community challenge at https://www.synapse.org/#!Synapse:syn11944948 (Choobdar *et al.*, 2019), and the Mouse Gene Informatics database (data retrieved on 2018-10-01, MGI) (Smith *et al.*, 2018).

Pre-processing genesets based on specificity, redundancy, and multi-functionality

Each of these six geneset-collections contained anywhere from about a hundred to tens of thousands of genesets (Table S2) that varied widely in specificity and redundancy. The first pre-processing step we did after downloading the data was to convert the original gene/protein IDs to entrez gene IDs, which was done using gene ID conversions found in MyGene.info (Wu *et al.*, 2013; Xin *et al.*, 2016). If the original ID mapped to more than one entrez ID, all of them were included for further analysis. Next, whenever applicable, annotations to genesets corresponding to terms in a curated ontology were propagated along the *is_a* and *part_of* relationships to ancestor terms in the corresponding ontologies: Gene Ontology (Ashburner *et al.*, 2000) for GOBP, Disease Ontology (Schriml *et al.*, 2019) for DisGeNet and BeFree, and Mammalian Phenotype Ontology (Smith and Eppig, 2009) for MGI.

Subsequent preprocessing steps were designed to ensure that the final set of genesets from each source are specific, largely non-overlapping, and not driven by multi-attribute genes.

Specificity: To select specific biologically-meaningful genesets in each collection, we sorted all the genesets in a collection from the largest to smallest based on the number of annotated genes (geneset size), manually examined their descriptions, and chose a size threshold that roughly separated large, generic genesets from the smaller, specific ones. This threshold was 200 for GOBP and KEGGBP, 300 for MGI, 400 for BeFree, 500 for GWAS and GOBPtmp, and 600 for DisGeNet.

Redundancy: To remove redundant genesets within a collection, first, we calculated the Jaccard index ($|A \cap B| / |A \cup B|$) and the overlap index ($|A \cap B| / min(|A|, |B|)$) between all pairs of genesets (with A and B representing the sets of genes annotated to the genesets). Then, we built a graph with the genesets as the nodes, and added edges between genesets pairs if their Jaccard index was >0.5 *and* their overlap index was >0.7. The geneset graph constructed in this manner contained many connected components, each representing a set of highly overlapping genesets. Finally, we used the following procedure to pick representative genesets within each component: a) calculate a score for each geneset equal to the sum of the proportions of genes in other linked genesets that are contained within it (higher this score, more representative that geneset is), b) create a sorted list of all the genesets in decreasing order of this score, and c) pick the first geneset in the list, remove every subsequent geneset that is connected to it in the graph, and

repeat this step until the sorted list is empty. This procedure resulted in a reasonable number of non-redundant genesets within each collection. The same Jaccard and overlap thresholds were used for all collections except MGI. For MGI, since an overlap cutoff of 0.7 still resulted in thousands of genesets, it was lowered to 0.5.
Multi-attribute genes: Given the set of largely non-overlapping genesets in a collection, individual genes were removed from all genesets if they appeared in more than 10 genesets in that collection. This step ensures that the evaluations are not biased by multi-attribute genes that can potentially be easily predicted in a non-specific manner (Gillis and Pavlidis, 2011).

We also note that we did not include the cellular component (CC) or molecular function (MF) classes of the gene ontology as part of the function classification tasks because two genes that are annotated to the same CC or MF need not be related to each other functionally.

Table S2. Information on the geneset-collections. The last four columns reflect the fact each geneset-collection is slightly different for every network and these values are presented as either a range, a median value, or number of genes in a union across all networks used in this study.

Geneset Collection	Number of Genesets From Original Data	Number of Genesets After Redundant Genesets Removed	Number of Genesets After Holdout Preprocessing	Geneset Sizes	Median Geneset Size	Number of Genes from Union of all Genesets
GOBPtmp	11,574 to 754*	166	(115, 160)	(27, 452)	174	9464
GOBP	11,574	313	(84, 96)	(20, 181)	76	5301
KEGGBP	149	138	(63, 74)	(24, 181)	51	3454
DisGeNet	4030	334	(89, 104)	(21, 368)	67	4689
BeFree	2891	207	(49, 57)	(20, 223)	80	2692
GWAS	169	74	(30, 37)	(24, 431)	94	2134
MGI	10,264	492	(90, 121)	(20, 132)	41	2716

* The GOBP temporal holdout step had an extra initial preprocessing step to make sure there were at least ten genes in the training and testing sets.

Calculating the network properties of the genesets

To determine how the performance of a given geneset depends on the network, for each geneset we calculated three different properties:

1) For a given geneset, T, the number of genes annotated it is given by $|T|$.
2) For a given geneset, T, the edge density, D_T, is given by

$$D_T = \sum_{(u,v)\in T} W_{uv} / (|T| * (|T| - 1)/2)\,, \quad \text{(eqn. S1)}$$

where W_{uv} is the edge weight between genes u and v. The edge density is a measure of how tightly connected the geneset is within itself.

3) For a given geneset, T, the segregation, S_T, is given by

$$S_T = \sum_{(u,v)\in T} W_{uv} / \sum_{u\in T,\, t\in V} W_{ut}\,. \quad \text{(eqn. S2)}$$

Segregation is a measure of how isolated the geneset is from the rest of the network.

The three geneset properties are shown for all geneset-collection–network combinations in Fig. S3. In general, there is little difference in the number of genes across the different prediction tasks (i.e. function, disease and trait), except for GOBPtmp, which has the largest number of genes due to the fact the genesets need to be larger to have enough with at least 10 testing genes. Edge density and segregation are highest for the function genesets (GOBPtmp, GOBP, KEGGBP) and lowest for the disease and trait genesets (DisGeNet, BeFree, GWAS, MGI).

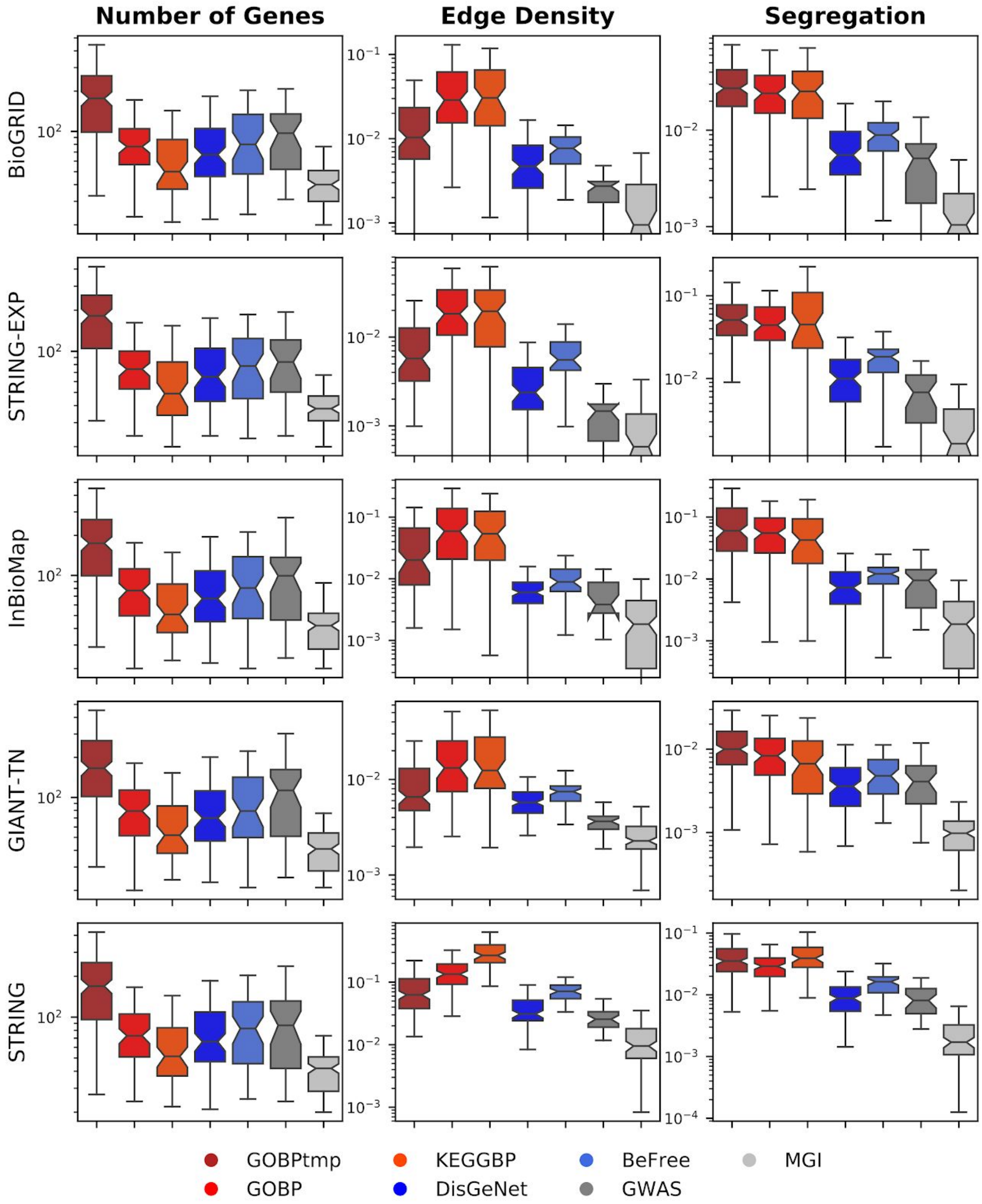

Fig. S3. Network properties for the different geneset-collections. The geneset-collections can be broken up into three prediction tasks; function (GOBPtmp, GOBP, KEGGBP; reds), disease (DiGeNet, BeFree; blues) and trait (GWAS, MGI; greys). In general, there is little difference in the number of genes across the different type prediction tasks (i.e. function, disease and trait), except for GOBPtmp which has the largest number of genes due to the fact the genesets need to be

larger to have enough with at least 10 testing genes. Edge density and segregation are highest for the function genesets (GOBPtmp, GOBP, KEGGBP) and lowest for the disease and trait genesets (DisGeNet, BeFree, GWAS, MGI).

Section 1.4: Validation schemes

We used three different validation schemes to evaluate gene classification.

Temporal holdout validation

Temporal holdout is the most stringent evaluation scheme for gene classification since it mimics the practical scenario of using current knowledge to predict the future. Since Gene Ontology was the only source with clear date-stamps for all its annotations, temporal holdout was applied only to the GOBP geneset-collection. Since the goal of this study is to use relatively recent and widely-used molecular networks, as this would reflect how these models would be deployed in practice, we chose a temporal cutoff point of Jan 1st, 2017. Then, for each geneset-collection, genes that only had an annotation to any geneset in the collection after 2017-01-01 were assigned to the testing set and the remaining genes were assigned to the training set. Since this resulted in the testing set having far fewer genes than the testing set for the other validation schemes, we made the following minor modifications to the geneset pre-processing procedure: GOBP geneset-collection was first filtered to remove any geneset with fewer than ten training genes or had fewer than ten testing genes based on the temporal split and the specificity threshold (maximum number of genes annotated to a geneset) was increased from 200 to 500. Redundancy filtering and multi-attribute gene filtering were unchanged. As noted in Section 1.1, from each network resource considered in this study, we chose the most recent version of the network that was released before 2017-01-01 to ensure no data leakage. Finally, genes were removed from genesets if they were not present in a given network, genesets with fewer than ten training genes or fewer than ten testing genes were filtered out, and the remaining genesets were used to perform the temporal holdout validation.

Study-bias holdout validation

The goal of study-bias validation is to evaluate the scenario that is close to the real-world situation of learning from well-characterized genes to predict novel un(der)-characterized genes. Here, we defined study-bias for each gene as the number of articles in PubMed (http://www.pubmed.gov/) in which that gene was referenced in, as determined in the *gene2pubmed* file (downloaded on 2018-10-30) from the NCBI Gene database (Brown *et al.*, 2015). Using this definition, for each geneset-collection–network combination, we created training-testing splits in the following manner: Genes were removed from genesets if they were not present in the given network. Then, among the remaining genes, a gene was assigned to the training set if it was in the top two-thirds of the list of genes sorted by their PubMed count. The remaining genes were assigned to the testing set. Finally, genesets with fewer than ten training genes or fewer than ten testing genes were filtered out and the remaining genesets were used to perform the study-bias holdout validation.

Five-fold cross-validation

To ensure comparability, we performed 5-fold cross-validation using the same genesets that were used in study-bias holdout, splitting each geneset randomly into five approximately equal folds (with similar proportions of positive and negative examples) and, in rotation, using one fold as the testing set and the remaining four as the training set.

Section 1.5: Evaluation Metrics

In this study, we present results in terms of two popular metrics, auPRC and auROC, as well as the precision of the top K ranked predictions (P@TopK). Since, each geneset-collection–network combination has a different number of positive examples (and, hence, different positive:negative proportions), we normalized auPRC and P@topK by the prior. Specifically, auPRC is given by:

$$auPRC = \log_2\left(\frac{auPRC_S}{prior} \right) \quad \text{(eqn. S3)}$$

where $auPRC_S$ is the standard area under the precision-recall curve, and the *prior* is *P*/(*P*+*N*) with *P* being the number of positive ground truth labels, and *N* being the number of negative ground truth labels. The log2 in eqn. S3 allows for the following interpretation: the number of 2-fold increases of the measured $auPRC_S$ over what is expected given the ground truth labels (e.g., a value of 1 indicates a 2-fold increase, a value of 2 indicates a 4-fold increase). Similarly, P@topK is given by:

$$P@topK = \log_2\left(\frac{TP_K}{K \times prior} \right) \quad \text{(eqn. S4)}$$

where K is the number of top-predictions to consider, TP_K is the number true-positives of the top-K predictions, and the *prior* is the same as in eqn. S3. We set K to be the number of ground truth positives in the testing set. P@topK can be thought of as what is the 2-fold increase in the percent of the top-K predictions that were predicted true over the expected value. Of note, it is possible that $TP_k = 0$ if no true positive is captured within the first *K* predictions. This causes P@topK to become $-\infty$. To address this issue, we set such values to be the minimum score obtained across all predictions for that given geneset-collection–network combination.

Precision-based metrics – auPRC and P@topK – are more suitable than the more popular area under the receiver-operating characteristic curve (auROC) for two reasons. First, gene classification is a highly imbalanced problem with many more negative examples than positive examples, and auROC is ill-suited for imbalanced problems (Saito and Rehmsmeier, 2015). Second, precision can control for Type-1 error (false positives) (Davis and Goadrich, 2006). Since the foremost reason for gene classification is to provide a list of candidate genes for further experimental study, it is more important to make sure the top predictions are as correct as possible, as opposed to ensuring that, on average, positive examples are ranked higher than negative examples. However, for completeness, we have provided auROC results in this Supplemental Material (Section 2).

Section 2: Supplementary Results

Section 2.1: Compiled results from all validation schemes in terms of all evaluation metrics

In this section, we present results for the ranking analysis used in Fig. 2, significance test analysis used in Fig. 3, as well as the boxplots representations seen in Fig. 4, based on all evaluation metrics (auPRC, P@TopK, and auROC) as well as all validation schemes (temporal holdout, study-bias holdout and 5FCV) (Figs. S4 - S8). Additionally, in this section, we present the results of how the performance of SL-A and LP-I scale with the number of genes, edge density, and segregation for all networks used in this study (Fig. S9).

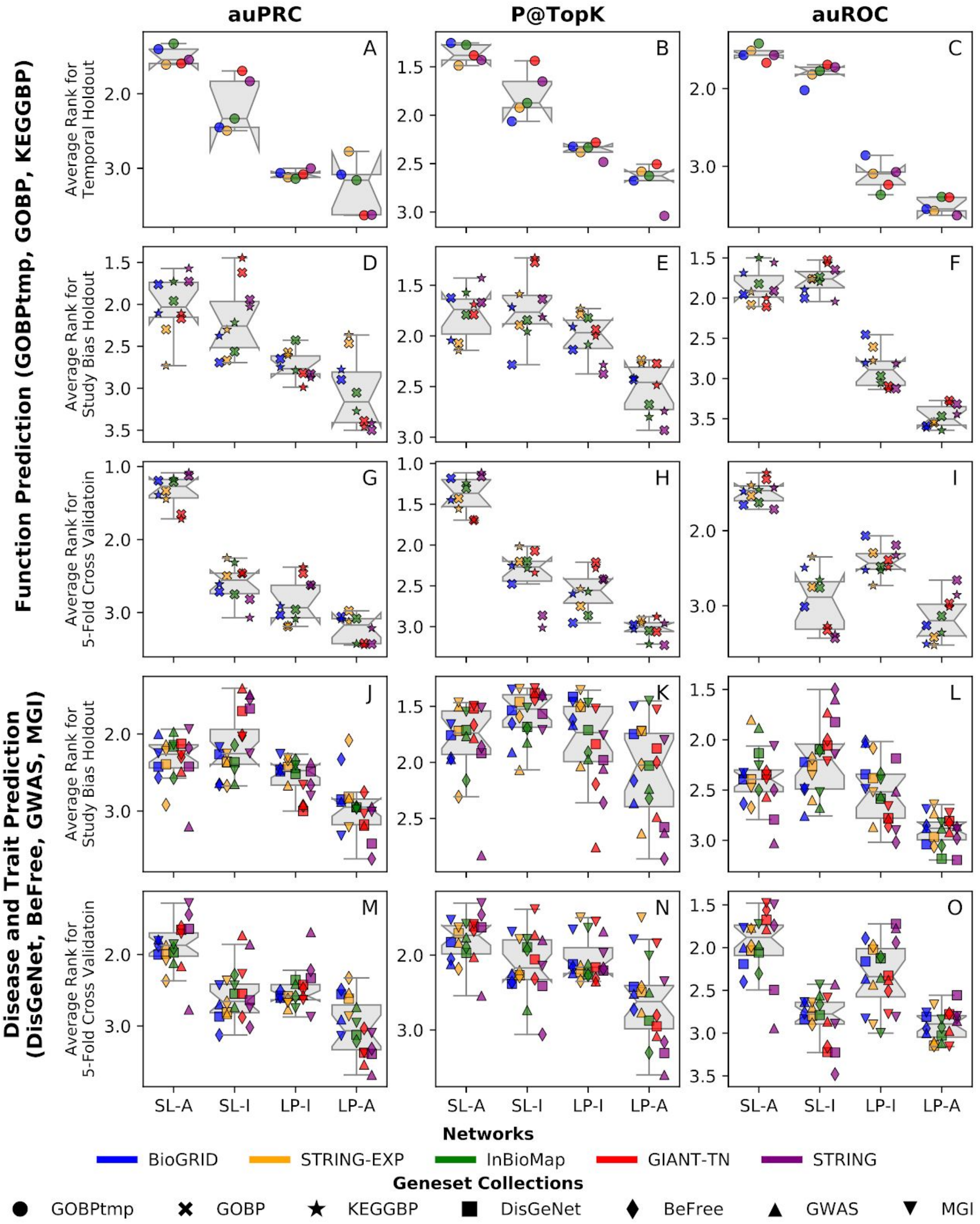

Fig. S4. Average rank of the four methods for all evaluation metrics and validation schemes. Each point in a boxplot represents the average rank for a geneset-collection–network combination, where the four methods were ranked in terms of performance for each geneset in a geneset-collection using the standard competition ranking. Different colors represent different networks and different marker shapes represent different geneset-collections.

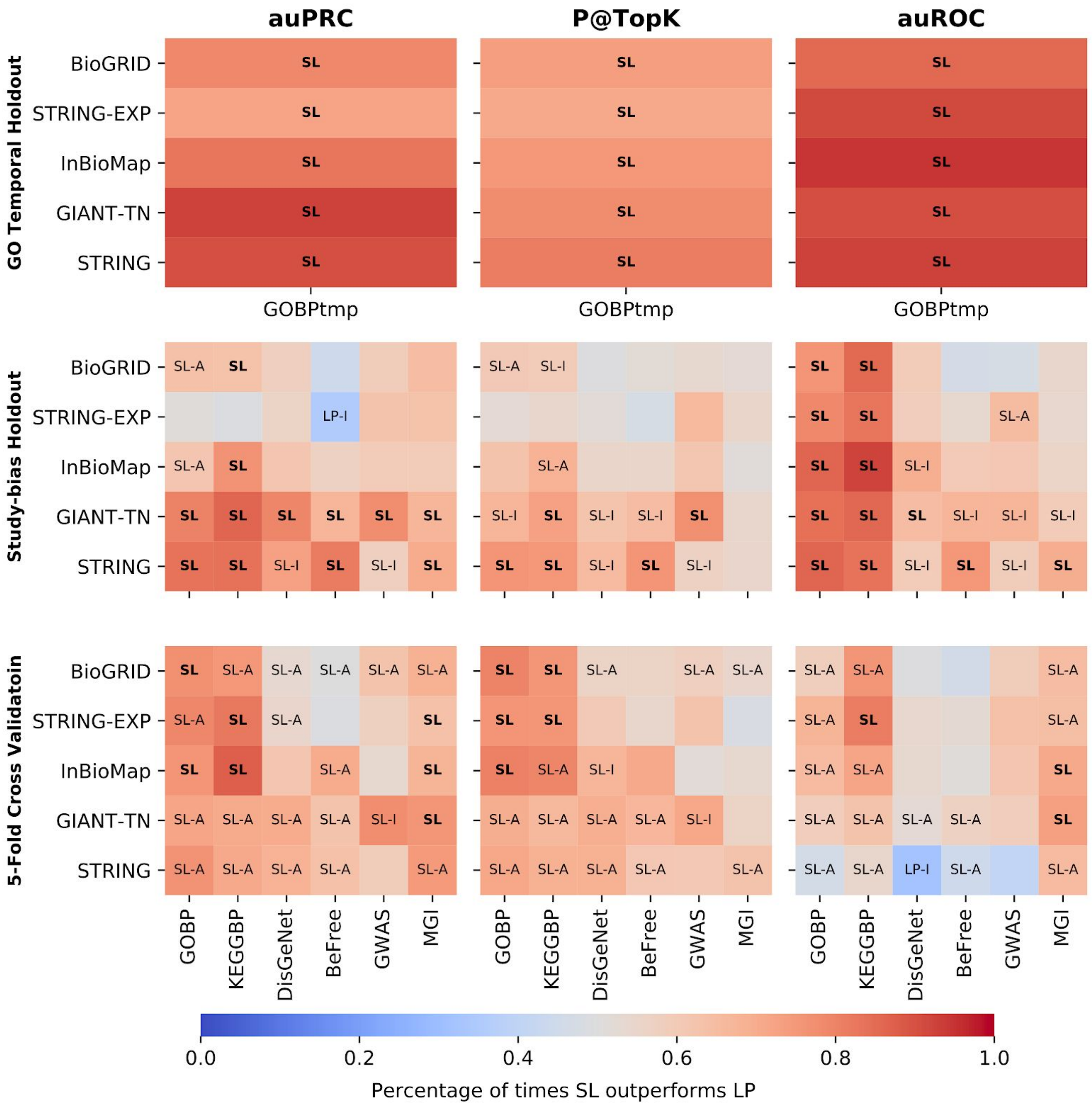

Fig. S5. Testing for a statistically significant difference between SL and LP methods using all evaluation metrics and validation schemes. For each network-geneset combination, each method is compared to the two methods from the other class (i.e. SL-A vs LP-I, SL-A vs LP-A, SL-I vs LP-I, SL-I vs LP-A). If a method was found to be significantly better than both methods from the other class (Wilcoxon ranked-sum test with an FDR threshold of 0.05), the cell is annotated with that method. If both models in that class were found to be significantly better than the two methods in the other class, the cell is annotated in bold with just the class. The color scale represents the fraction of genesets that were higher for the SL methods across all four comparisons. The first column uses GOBP temporal holdout, whereas the remaining 6 columns use study-bias holdout. B) SL methods show a statistically significant improvement over LP methods, especially for function prediction.

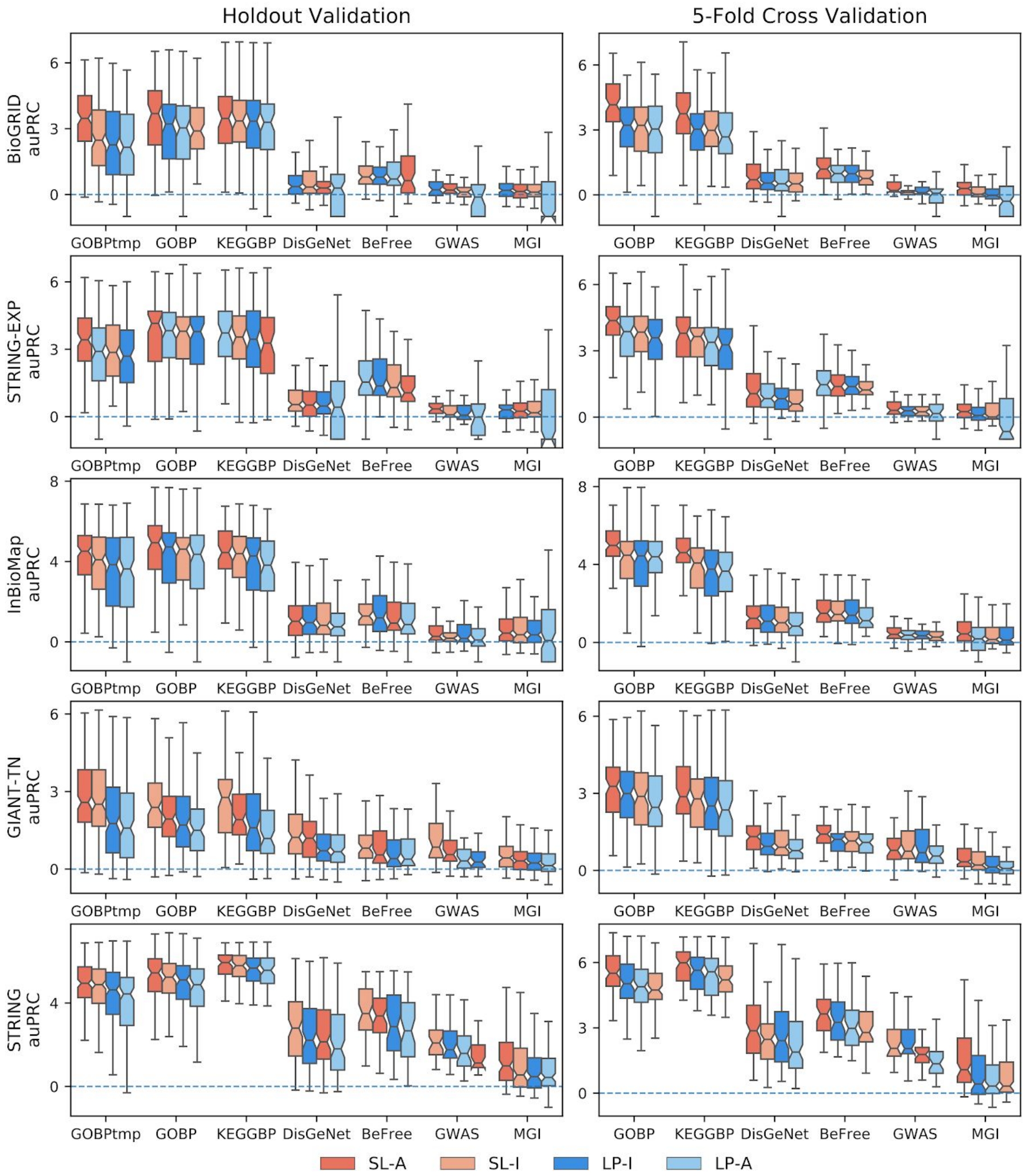

Fig. S6. Boxplots for auPRC performance across all geneset-collection–network combinations. The performance for each individual geneset-collection–network combination is compared across the four methods; SL-A (red), SL-I (light red), LP-I (blue), and LP-A (light blue). The methods are ranked by median value with the highest scoring method on the left. The first column contains temporal and study-bias holdout, and the second column is 5FCV. The scoring metric is auPRC.

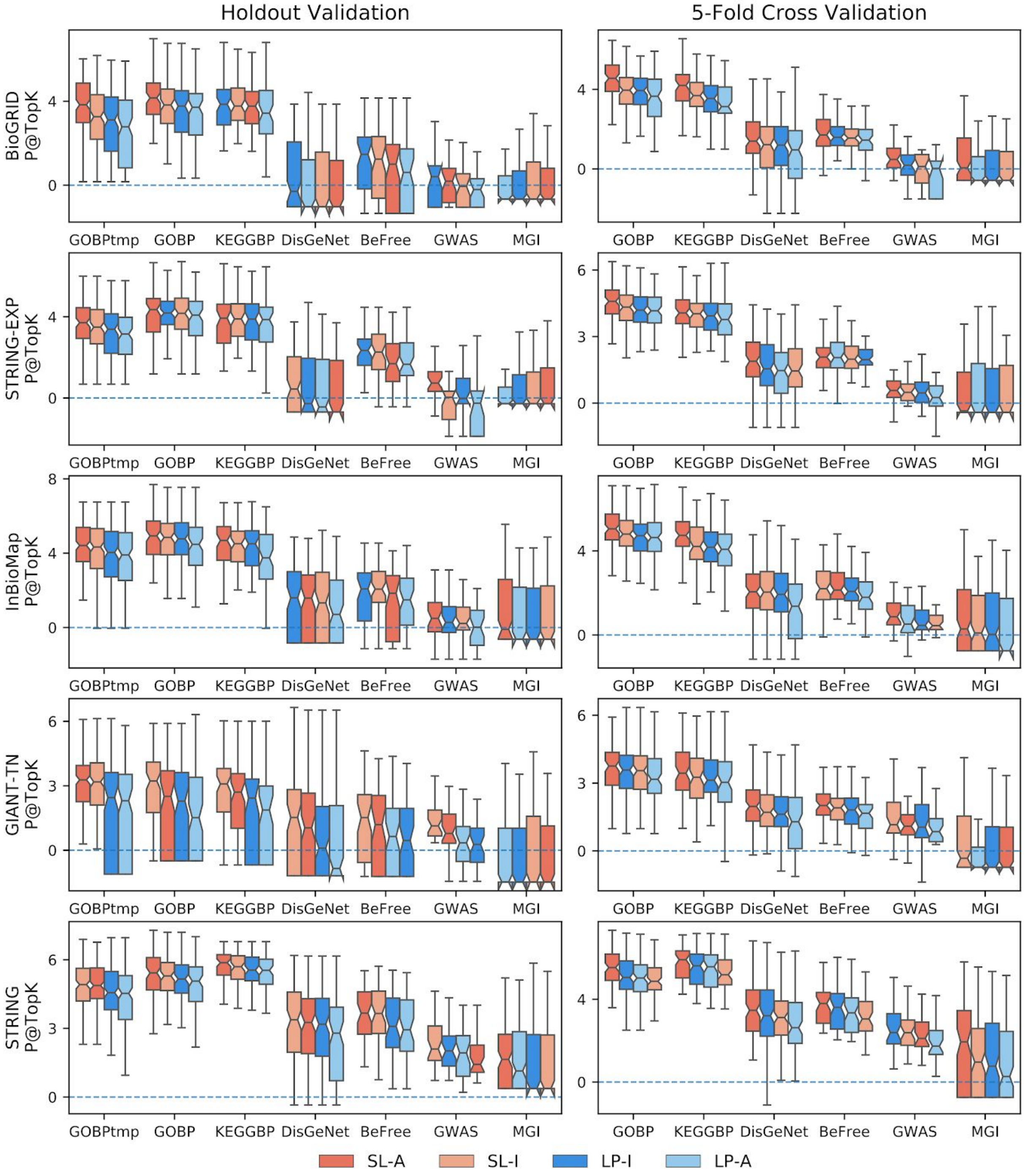

Fig. S7. Boxplots for P@TopK performance across all geneset-collection–network combinations. The performance for each individual geneset-collection–network combination is compared across the four methods; SL-A (red), SL-I (light red), LP-I (blue), and LP-A (light blue). The methods are ranked by median value with the highest scoring method on the left. The first column contains temporal and study-bias holdout, and the second column is 5FCV. The scoring metric is P@TopK.

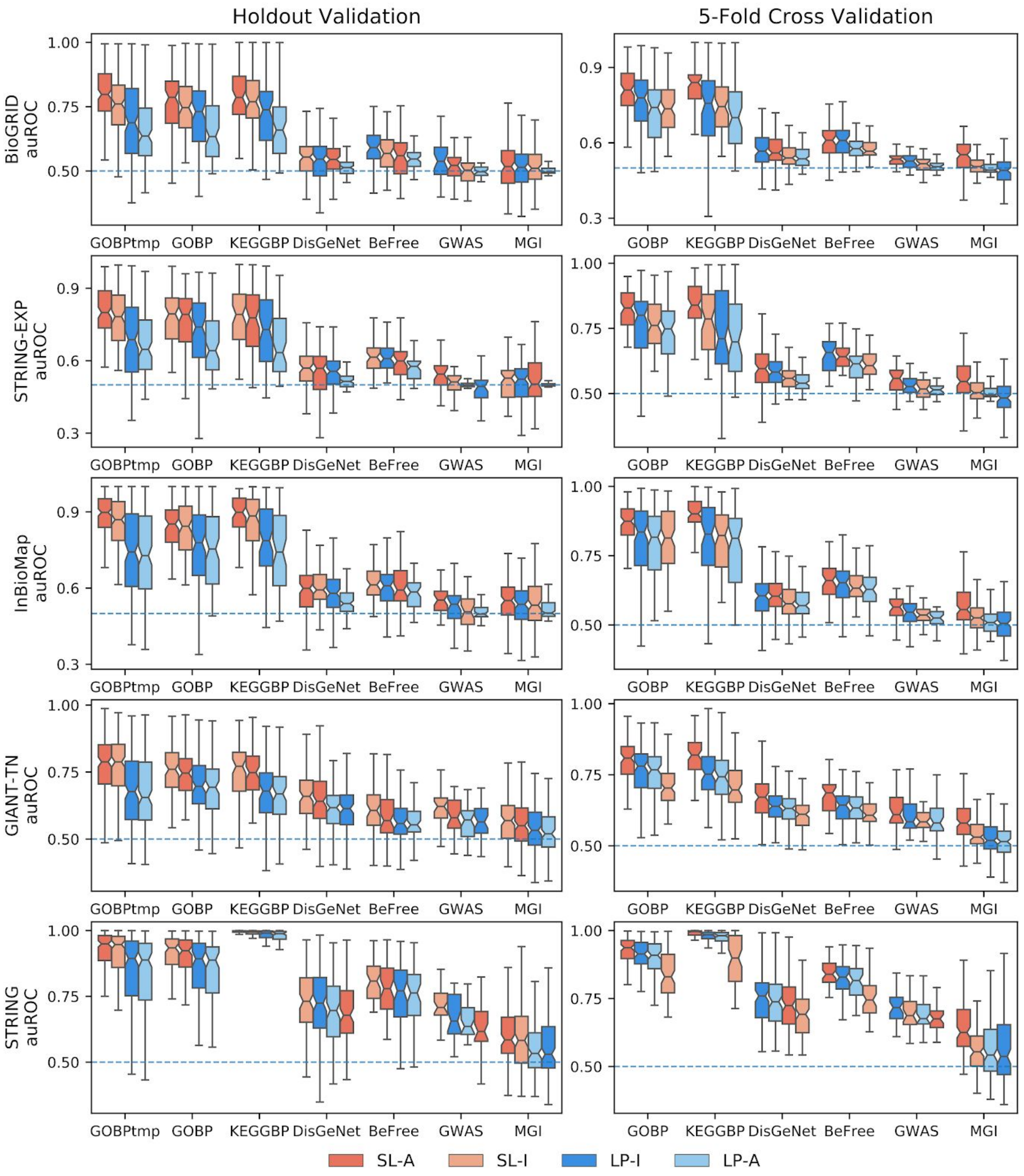

Fig. S8. Boxplots for auROC performance across all geneset-collection–network combinations. The performance for each individual geneset-collection–network combination is compared across the four methods; SL-A (red), SL-I (light red), LP-I (blue), and LP-A (light blue). The methods are ranked by median value with the highest scoring method on the left. The first column contains temporal and study-bias holdout, and the second column is 5FCV. The scoring metric is auROC.

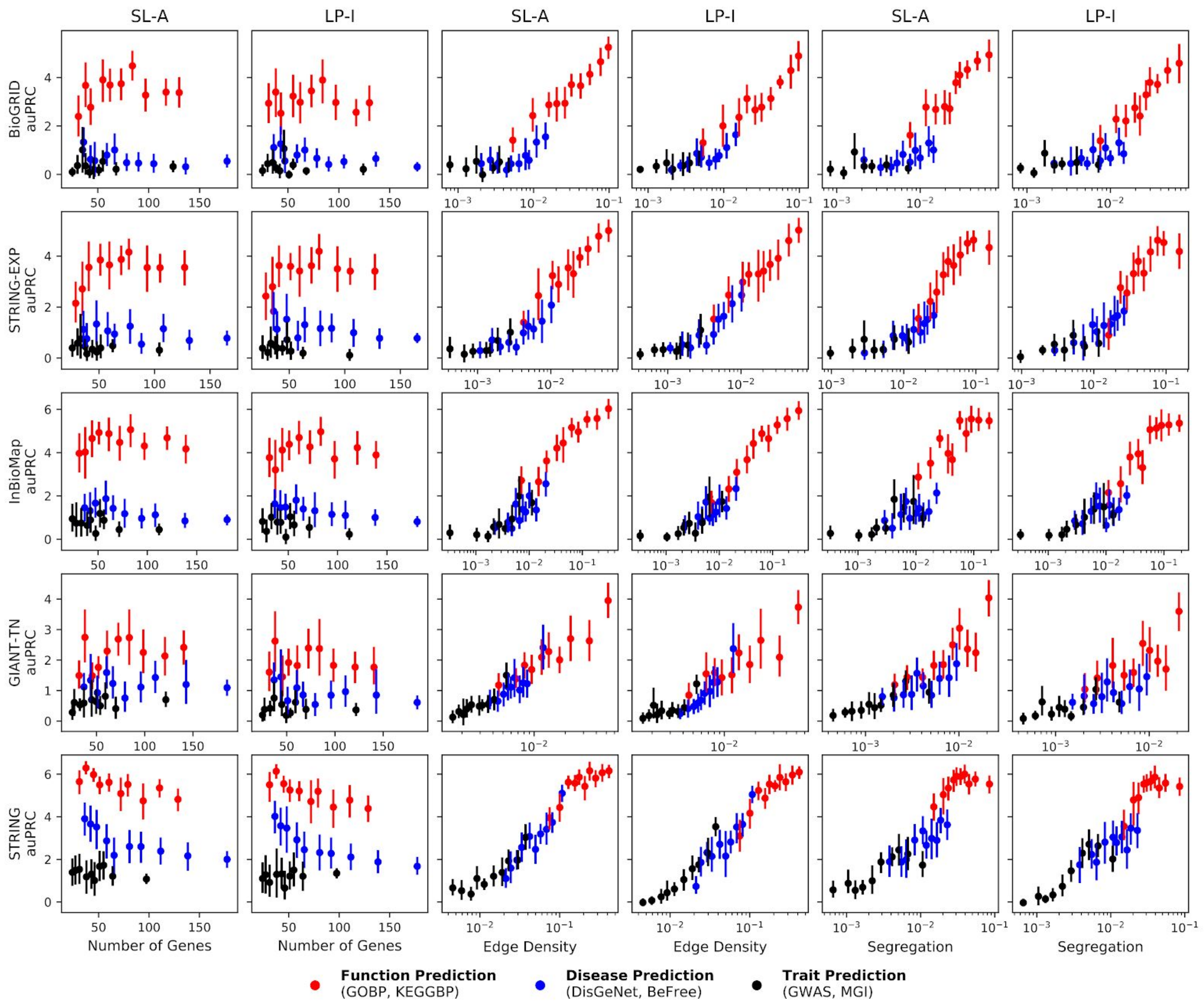

Fig S9. Performance vs Network/Geneset properties for all networks. SL-A is able to capture network information as efficiently as LP-I across all networks. There is no correlation between the number of genes in the geneset versus performance, but there is a strong correlation between the performance and the edge density as well as segregation. The different colored dots represent function genesets (red, GOBP and KEGGBP), disease genesets (blue, DIsGeNet and BeFree), and trait genesets (black, GWAS and MGI). The vertical line is the 95% confidence interval and the performance metric is auPRC.

Section 2.2: Effect Size

In this section, we show results for the effect size between all methods (SL-A, SL-I, LP-I, LP-A). To calculate an effect size, for every geneset we calculate the ratio of auRPC values, find the percent increase/decrease and then take the median value for every geneset-collection–network combination. The results show that SL-A has a significant effect size when compared to LP-I for function prediction (53% for temporal holdout and 19% for Study-bias holdout). Also, for all prediction tasks the effect size seen between the SL methods and LP-I is equal to or greater than the effect size between LP-I and LP-A, where LP-I is widely considered a much better model than LP-A and thus, the comparison between LP-I and LP-A can be viewed as a baseline effect size.

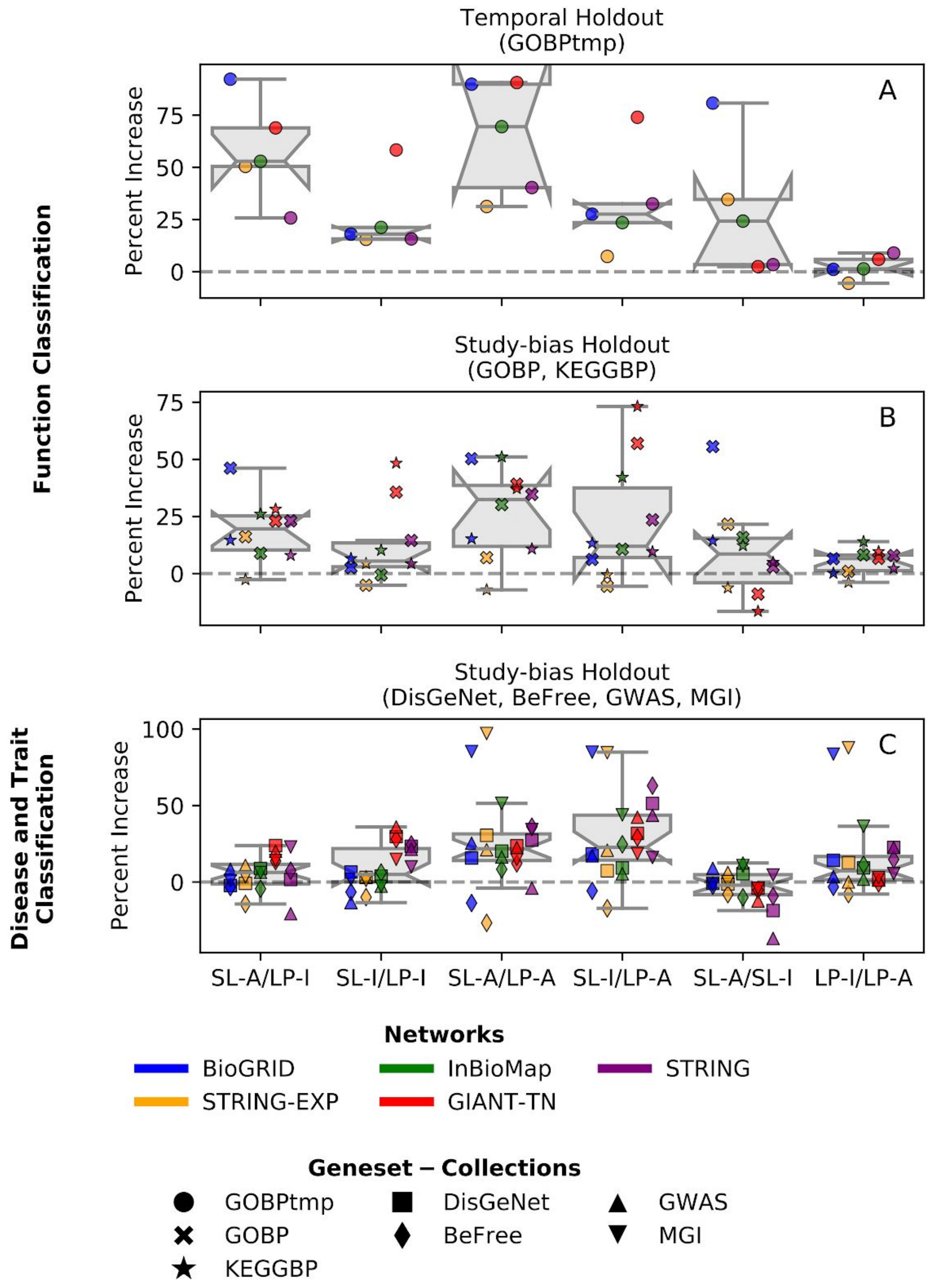

Fig S10. Effect size for every pair of methods. Each point is the median percent increase for every geneset-collection–network combination. (A) Functional prediction tasks using GOBP temporal holdout, (B) Functional prediction tasks using study-bias holdout for GOBP and KEGGBP, and (C) Disease and trait prediction tasks using study-bias holdout for DisGeNet, BeFree, GWAS, and MGI. The results are shown for auPRC where different colors represent different networks and different marker styles represent the different geneset-collections.

Section 2.3: Label Propagation with Negative Examples

In this section, we show results for using negative examples in label propagation (LPN). We performed the same hyperparameter tuning for the restart parameter as described in Supplemental Section 1.2 and find a

restart parameter of 0.45 is optimum when using negative examples (Fig. S11). This optimal value for the restart parameter in LPN is relatively low compared to the optimum value for LP (expect for the GIANT-TN network were both LP and LPN prefer a higher restart value). It is worth noting, that just like with LP, the dependance on the restart parameter is minimal (Fig. S11B). We also include boxplots comparing label propagation with and without negative examples (Fig. S12). Lastly, we show a side by side comparison of the ranking analysis (Fig. S13) and Wilxcon analysis (Fig. S14) using label propagation with and without negative examples. The results show that even though using negative examples slightly increases performance in label propagation, the results when compared against supervised learning remain unchanged.

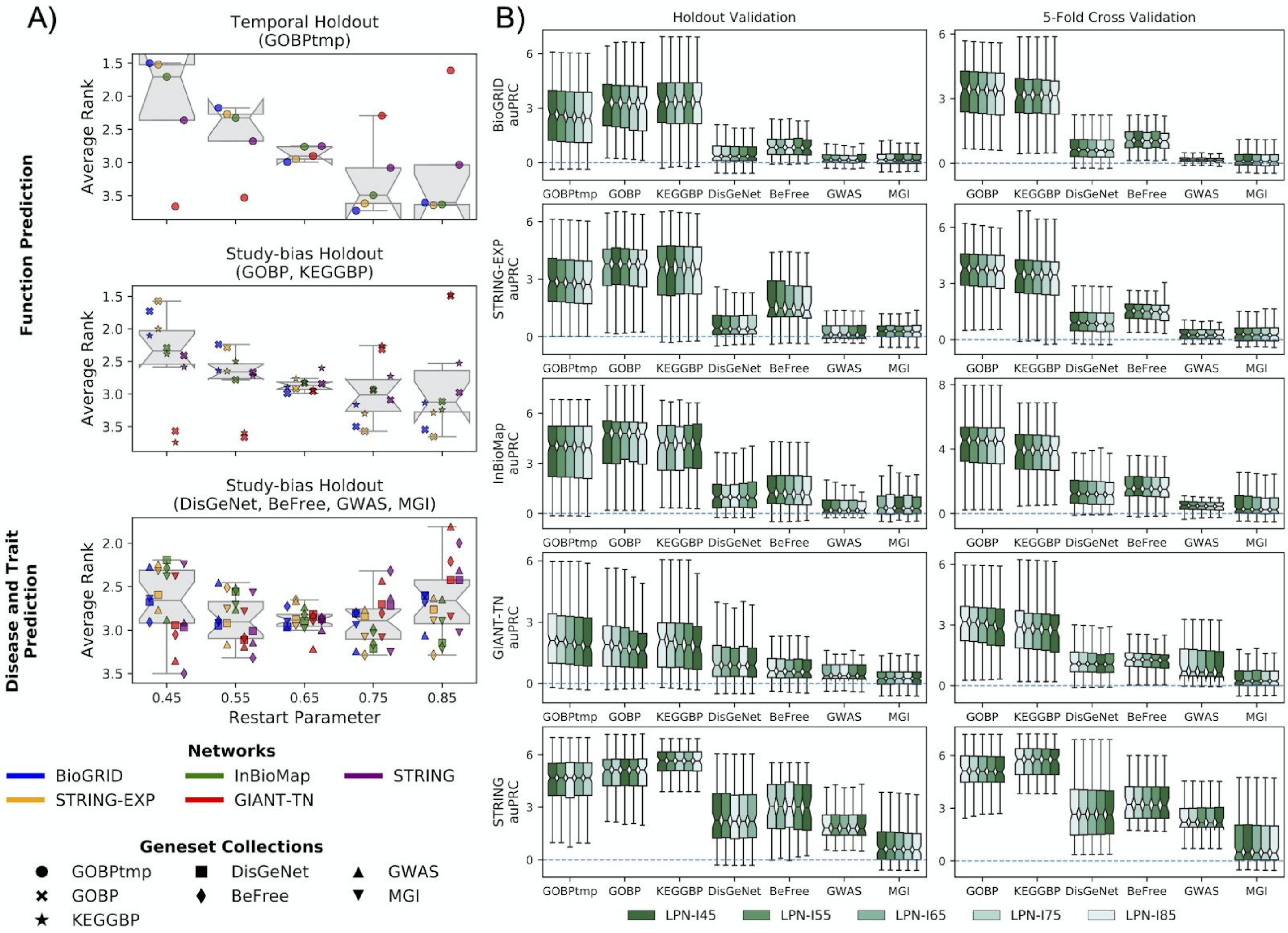

Fig. S11. Tuning the restart probability hyperparameter when using negative examples in label propagation. A) Each point in each boxplot represents the average rank for a geneset-collection–network combination, where the five restart probabilities (0.45, 0.55, 0.65, 0.75 and 0.85) were ranked in terms of performance (auPRC) for each geneset in a geneset-collection using the standard competition ranking. A restart probability of 0.45 was chosen as optimal. B) The performance for each individual geneset-collection–network combination is compared across the five restart probabilities. The methods are ranked by median value of auRPC with the highest scoring method on the left. There is no strong dependence of auRPC on the restart probability.

Fig. S12. Boxplots for performance across all geneset-collection–network combinations for label propagation on the influence matrix with and without using negative examples. A) The performance for each individual geneset-collection–network combination is compared for label propagation with negative examples (LPN-I, blue) and label propagation without negative examples (LP-I, green). The methods are ranked by median value with the highest scoring method on the left. Results show LPN-I has a moderately increased performance when compared to LP-I. B) Each point in the plot is the median value from one of the boxplots in A. This shows that both LPN and LP methods perform better for function prediction compared to disease/trait prediction.

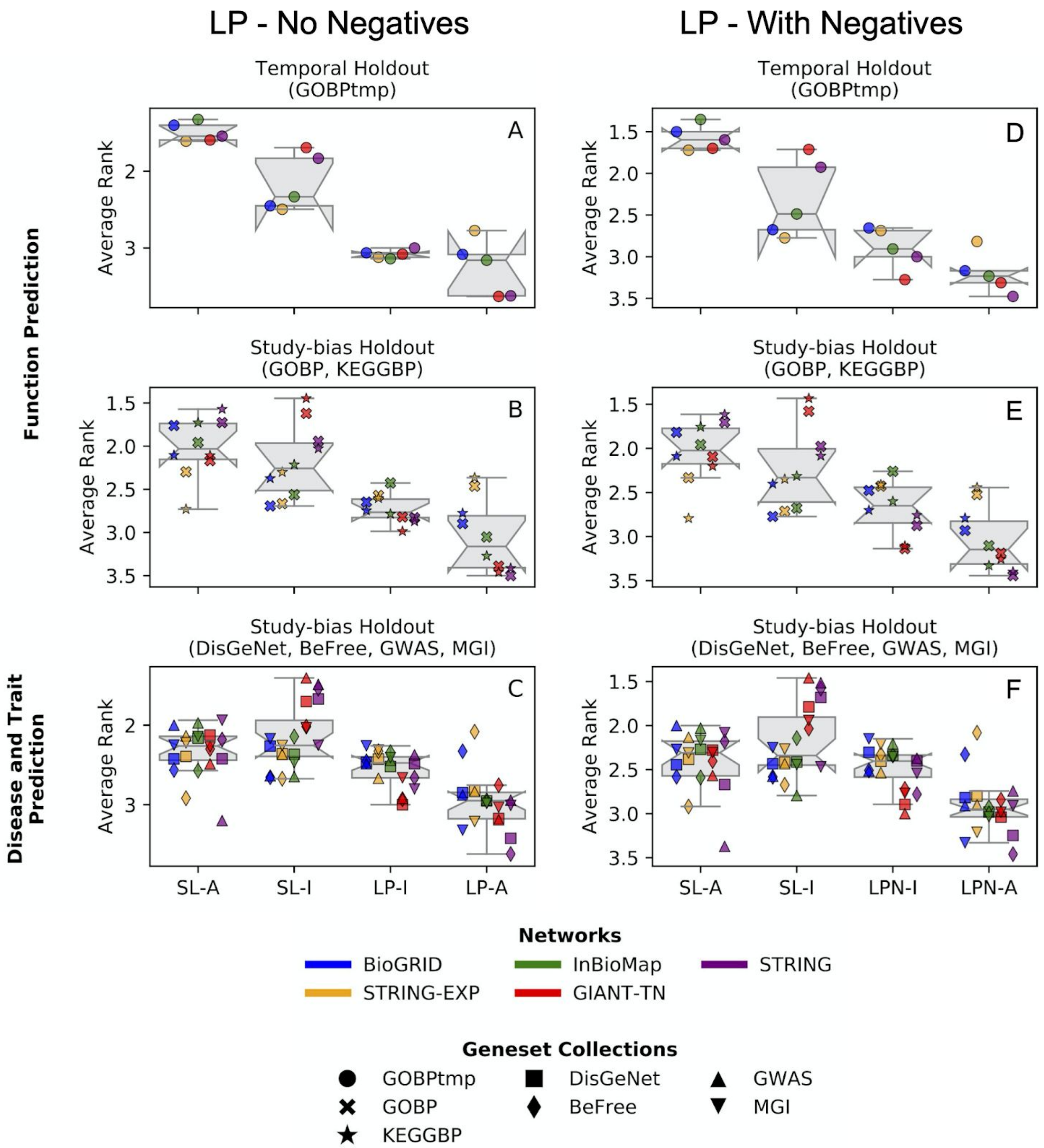

Fig. S13. Comparing results from average rank analysis with and without using negative examples in label propagation. The left column has label propagation without negative examples (LP) and the right column has label propagation with negative examples (LPN). Each point in each boxplot represents the average rank for a geneset-collection–network combination, obtained based on ranking the four methods in terms of performance for each geneset in a geneset-collection using the standard competition ranking. (A, D) Functional prediction tasks using GOBP temporal holdout, (B, E) Functional prediction tasks using study-bias holdout for GOBP and KEGGBP, and (C, F) Disease and trait prediction tasks using study-bias holdout for DisGeNet, BeFree, GWAS, and MGI. The results are shown for auPRC where different colors represent different networks and different marker styles represent the different geneset-collections. The results show that no substantial difference can be seen between using or not using negative examples in label propagation.

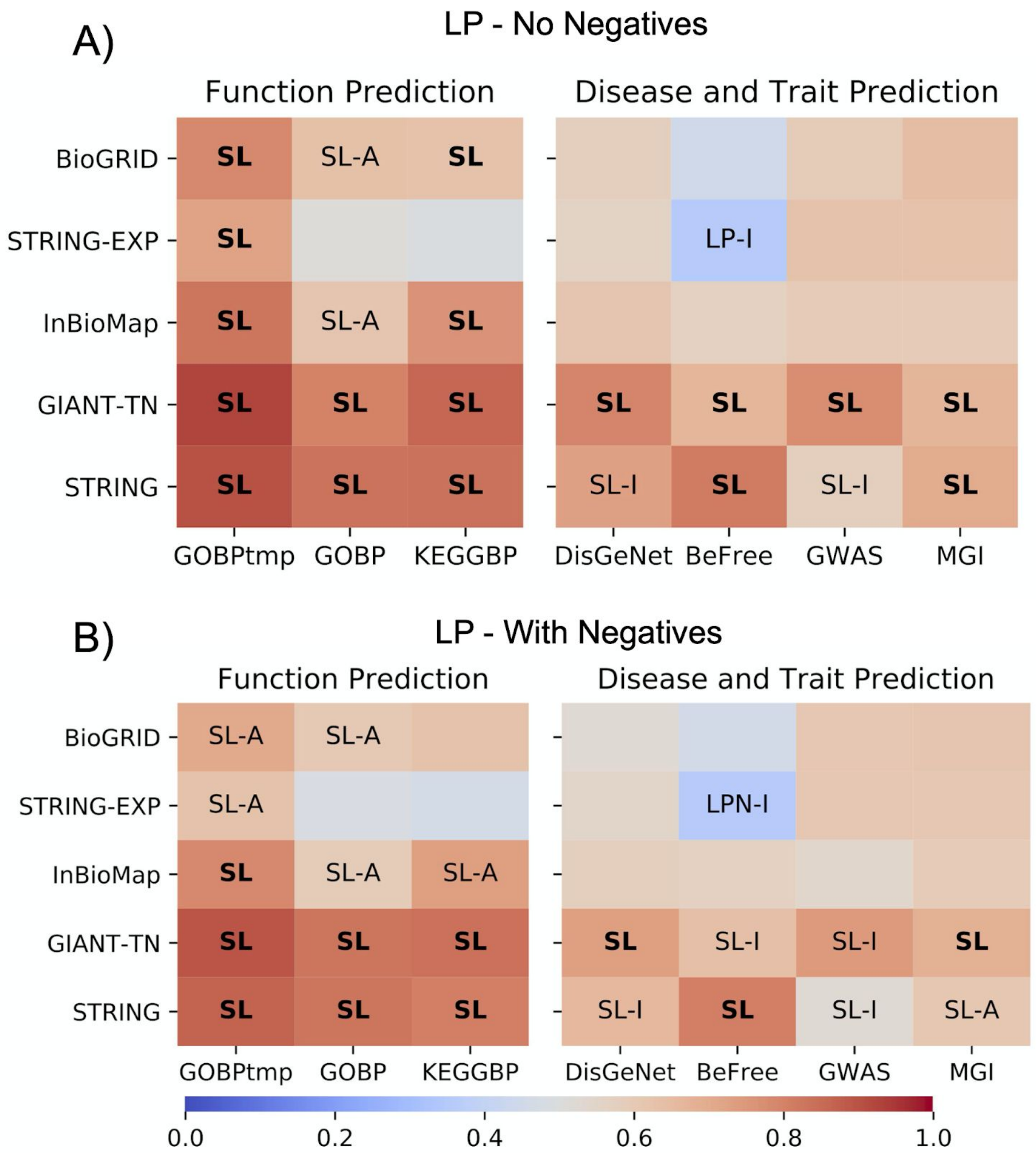

Fig. S14. Comparing the Wilcoxon statistical test analysis with and without using negative examples in label propagation. A) Label propagation without negative examples (LP) and B) label propagation with negative examples (LPN). For each network-geneset combination, each method is compared to the two methods from the other class. If a method was found to be significantly better than both methods from the other class (Wilcoxon ranked-sum test with an FDR threshold of 0.05), the cell is annotated with that method. If both models in that class were found to be significantly better than the two methods in the other class, the cell is annotated in bold with just the class. The color scale represents the fraction of genesets that were higher for the SL methods across all four comparisons. The first column uses GOBP temporal holdout, whereas the remaining 6 columns use study-bias holdout. The results show that no substantial difference can be seen between using or not using negative examples in label propagation.

References

Ashburner,M. *et al.* (2000) Gene ontology: tool for the unification of biology. The Gene Ontology Consortium. *Nat. Genet.*, **25**, 25–29.

Brown,G.R. *et al.* (2015) Gene: a gene-centered information resource at NCBI. *Nucleic Acids Res.*, **43**, D36–D42.

Choobdar,S. *et al.* (2019) Open Community Challenge Reveals Molecular Network Modules with Key Roles in Diseases. *bioRxiv*, 265553.

Davis,J. and Goadrich,M. (2006) The Relationship Between Precision-Recall and ROC Curves. In, *Proceedings of the 23rd International Conference on Machine Learning*, ICML ’06. ACM, New York, NY, USA, pp. 233–240.

Gillis,J. and Pavlidis,P. (2011) The Impact of Multifunctional Genes on ‘Guilt by Association’ Analysis. *PLOS ONE*, **6**, e17258.

Goyal,P. and Ferrara,E. (2018) Graph Embedding Techniques, Applications, and Performance: A Survey. *Knowl.-Based Syst.*, **151**, 78–94.

Greene,C.S. *et al.* (2015) Understanding multicellular function and disease with human tissue-specific networks. *Nat. Genet.*, **47**, 569–576.

Grover,A. and Leskovec,J. (2016) node2vec: Scalable Feature Learning for Networks. In, *Proceedings of the 22nd ACM SIGKDD International Conference on Knowledge Discovery and Data Mining - KDD ’16*. ACM Press, San Francisco, California, USA, pp. 855–864.

Huang,J.K. *et al.* (2018) Systematic Evaluation of Molecular Networks for Discovery of Disease Genes. *Cell Syst.*, **6**, 484-495.e5.

Kanehisa,M. *et al.* (2017) KEGG: new perspectives on genomes, pathways, diseases and drugs. *Nucleic Acids Res.*, **45**, D353–D361.

Kanehisa,M. *et al.* (2019) New approach for understanding genome variations in KEGG. *Nucleic Acids Res.*, **47**, D590–D595.

Kanehisa,M. and Goto,S. (2000) KEGG: Kyoto Encyclopedia of Genes and Genomes. *Nucleic Acids Res.*, **28**, 27–30.

Li,T. *et al.* (2017) A scored human protein-protein interaction network to catalyze genomic interpretation. *Nat. Methods*, **14**, 61–64.

Pedregosa,F. *et al.* (2011) Scikit-learn: Machine Learning in Python. *J. Mach. Learn. Res.*, **12**, 2825–2830.

Piñero,J. *et al.* (2017) DisGeNET: a comprehensive platform integrating information on human disease-associated genes and variants. *Nucleic Acids Res.*, **45**, D833–D839.

Piñero,J. *et al.* (2015) DisGeNET: a discovery platform for the dynamical exploration of human diseases and their genes. *Database*, **2015**.

Saito,T. and Rehmsmeier,M. (2015) The Precision-Recall Plot Is More Informative than the ROC Plot When Evaluating Binary Classifiers on Imbalanced Datasets. *PLOS ONE*, **10**, e0118432.

Schriml,L.M. *et al.* (2019) Human Disease Ontology 2018 update: classification, content and workflow expansion. *Nucleic Acids Res.*, **47**, D955–D962.

Smith,C.L. *et al.* (2018) Mouse Genome Database (MGD)-2018: knowledgebase for the laboratory mouse. *Nucleic Acids Res.*, **46**, D836–D842.

Smith,C.L. and Eppig,J.T. (2009) The mammalian phenotype ontology: enabling robust annotation and comparative analysis. *Wiley Interdiscip. Rev. Syst. Biol. Med.*, **1**, 390–399.

Stark,C. *et al.* (2006) BioGRID: a general repository for interaction datasets. *Nucleic Acids Res.*, **34**, D535–D539.

Szklarczyk,D. *et al.* (2015) STRING v10: protein-protein interaction networks, integrated over the tree of life. *Nucleic Acids Res.*, **43**, D447-452.

The Gene Ontology Consortium (2019) The Gene Ontology Resource: 20 years and still GOing strong. *Nucleic Acids Res.*, **47**, D330–D338.

Wu,C. *et al.* (2013) BioGPS and MyGene.info: organizing online, gene-centric information. *Nucleic Acids Res.*, **41**, D561–D565.

Xin,J. *et al.* (2016) High-performance web services for querying gene and variant annotation. *Genome Biol.*, **17**, 91.
